# Agreement between Computerized and Human Assessment of Performance on the Ruff Figural Fluency Test

**DOI:** 10.1371/journal.pone.0163286

**Published:** 2016-09-23

**Authors:** Martin F. Elderson, Sander Pham, Marlise E. A. van Eersel, LifeLines Cohort Study, Bruce H. R. Wolffenbuttel, Johan Kok, Ron T. Gansevoort, Oliver Tucha, Melanie M. van der Klauw, Joris P. J. Slaets, Gerbrand J. Izaks

**Affiliations:** 1 University of Groningen, University Medical Center Groningen, Department of Endocrinology, Groningen, the Netherlands; 2 University of Groningen, The Lifelines Cohort Study, Groningen, the Netherlands; 3 University of Groningen, University Medical Center Groningen, University Center for Geriatric Medicine, Groningen, the Netherlands; 4 University of Groningen, University Medical Center Groningen, Department of Nephrology, Groningen, the Netherlands; 5 University of Groningen, Department of Clinical and Developmental Neuropsychology, Groningen, the Netherlands; 6 Leyden Academy on Vitality and Ageing, Leyden, the Netherlands; University of Zurich, SWITZERLAND

## Abstract

The Ruff Figural Fluency Test (RFFT) is a sensitive test for nonverbal fluency suitable for all age groups. However, assessment of performance on the RFFT is time-consuming and may be affected by interrater differences. Therefore, we developed computer software specifically designed to analyze performance on the RFFT by automated pattern recognition. The aim of this study was to compare assessment by the new software with conventional assessment by human raters. The software was developed using data from the Lifelines Cohort Study and validated in an independent cohort of the Prevention of Renal and Vascular End Stage Disease (PREVEND) study. The total study population included 1,761 persons: 54% men; mean age (SD), 58 (10) years. All RFFT protocols were assessed by the new software and two independent human raters (criterion standard). The mean number of unique designs (SD) was 81 (29) and the median number of perseverative errors (interquartile range) was 9 (4 to 16). The intraclass correlation coefficient (ICC) between the computerized and human assessment was 0.994 (95%CI, 0.988 to 0.996; p<0.001) and 0.991 (95%CI, 0.990 to 0.991; p<0.001) for the number of unique designs and perseverative errors, respectively. The mean difference (SD) between the computerized and human assessment was -1.42 (2.78) and +0.02 (1.94) points for the number of unique designs and perseverative errors, respectively. This was comparable to the agreement between two independent human assessments: ICC, 0.995 (0.994 to 0.995; p<0.001) and 0.985 (0.982 to 0.988; p<0.001), and mean difference (SD), -0.44 (2.98) and +0.56 (2.36) points for the number of unique designs and perseverative errors, respectively. We conclude that the agreement between the computerized and human assessment was very high and comparable to the agreement between two independent human assessments. Therefore, the software is an accurate tool for the assessment of performance on the RFFT.

## Introduction

Cognitive decline is a common chronic condition in old age. Worldwide, an estimated 36 million people live with dementia and it is expected that this number will double every twenty years to approximately 115 million in 2050 [1,2]. It is generally believed that dementia is the result of a long-term pathological process that spans at least two to three decades. This is supported by the recent finding that cognitive decline is already evident at the age of 45 years [3]. Therefore, cognitive decline is an important outcome in life course epidemiology and prospective cohort studies. However, few cognitive test are sensitive to cognitive changes across life span, from young adulthood to old age.

The Ruff Figural Fluency Test (RFFT) is a sensitive cognitive test for changes in nonverbal fluency suitable for all age groups [4–6]. The test measures the ability to draw as many unique designs as possible within a set time period. Performance on the RFFT is associated with various biological characteristics such as for example, frontal gray matter volume in Alzheimer's disease and right frontal delta magnitude on quantitative electroencephalography [7,8]. The test provides insight in many different cognitive abilities that range from initiation and planning to divergent reasoning and mental flexibility [4,5]. These characteristics and the limited time that is required to administer the test, make the RFFT a useful outcome measure for cognitive function. Therefore, the RFFT was introduced as a cognitive test in the Lifelines Cohort Study that included 167,729 participants of the general population [9]. However, the assessment of performance on the RFFT is time-consuming as the number of unique designs can be large and some designs can be complex and highly similar. These characteristics of the RFFT probably also increase the chance of errors and differences between raters. Moreover, assessment of a neuropsychological test as the RFFT requires expert knowledge and human raters have to be trained and supervised by a qualified neuropsychologist. This can be a burden on resources. To overcome these problems and to improve the usability of the RFFT in large sample studies, we developed a dedicated software program that was specifically designed to analyze performance on the RFFT by automated pattern recognition.

The aim of this study was to compare assessment of performance on the RFFT by the new software program with conventional assessment by human raters. The total study population included 1,761 community-dwelling persons aged 40–87 years. All RFFT protocols were assessed by the new software and two independent human raters.

## Methods

### Study population

The study population included 1,761 participants of the fifth survey of the Prevention of Renal and Vascular ENd-stage Disease study (PREVEND) who performed the RFFT. The PREVEND study was initiated in 1997 in the city of Groningen, the Netherlands, and designed to investigate prospectively the natural course of (micro)albuminuria and its relation to renal and cardiovascular disease in the general population [10,11]. The fifth survey of PREVEND was performed from 2009 to 2012.

### Ethics statement

The PREVEND study has been approved by the Medical Ethical Committee (METc) of the University Medical Center Groningen, Groningen, the Netherlands, and was conducted in accordance with the guidelines of the Declaration of Helsinki. Written informed consent was obtained from all participants. The authors MFE, MEAVE and GJI were involved with the collection of the data and had access to identifying information. The data were anonymized prior to analysis.

### Ruff Figural Fluency Test

**Fig 1:**
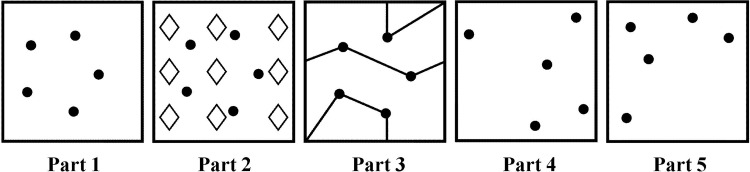
Five-dot patterns in parts 1 to 5 of the Ruff Figural Fluency Test. Each part consists of 35 identical five-dot patterns [4]. See also reference 6.

As described previously [6], the Ruff Figural Fluency Test (RFFT) [4,5] is a measure of nonverbal fluency consisting of five parts [5,12]. All parts (1 to 5) consist of 35 five-dot patterns arranged in seven rows and five columns on an 8.5 x 11” sheet of paper. However, the stimulus pattern differs between the parts (Fig 1). In part 1, the five-dot pattern forms a regular pentagon. In parts 2 and 3, the five-dot pattern of part 1 is repeated but includes various distractors: diamonds in part 2, and lines in part 3. In parts 4 and 5, the five-dot pattern is a variation of the pattern of part 1 and these parts do not contain distracting elements. In each part, the task is to draw as many unique designs as possible within one minute by connecting the dots in a different pattern. Repetitions of designs are scored as perseverative errors. Performance on the RFFT is expressed as the total number of unique designs (the sum of all five parts) and the total number of perseverative errors [5,12].

### Human assessment

Performance on the RFFT was analyzed independently by two trained raters (referred to as rater 1 and rater 2). The analysis was repeated by a third independent rater (rater 3) if the number of unique designs or perseverative errors as analyzed by the first two raters differed by more than two points in one part or more than four points in total. Then, for each participant, the RFFT scores as analyzed by the two raters who were most concordant were averaged. All raters were undergraduate students ranging in age from 18 to 22 years old. RFFT protocols were analyzed by different subsets of raters. The human assessment was defined as the criterion standard.

### Computerized assessment

All RFFT protocols were scanned in color with a Kodak i620 scanner at a resolution of 300 dots per inch and saved in portable network graphics (PNG) format. Subsequently, the RFFT protocols in PNG format were analyzed by the specifically designed software for the computerized assessment.

The software was developed using data from the Lifelines Cohort Study [9]. Lifelines is a multidisciplinary prospective population-based cohort study examining in a three-generation design the health and health-related behaviors of 167,729 persons living in the north of the Netherlands. It employs a broad range of investigative procedures in assessing the biomedical, socio-demographic, behavioral, physical and psychological factors which may contribute to health and disease of the general population, with a special focus on multimorbidity and complex genetics.

**Fig 2:**
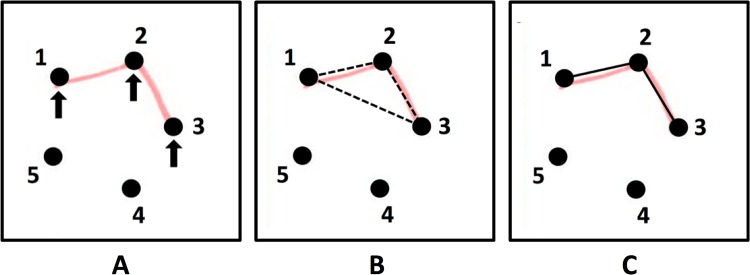
Illustration of active dots, candidate connections and true connections. A. Active dots are part of the design drawn by the respondent (red lines). Here, 1, 2, and 3 are active dots (arrows); 4 and 5 are inactive dots. B. Candidate connections are all connections that can possibly exist between the active dots (dashed black lines). C. True connections are connections that are compatible with the red lines drawn by the respondent: here, connections 1–2 and 2–3 (black lines).

Development of the software was based on the principle that in each cell of the standard RFFT protocol, no more than ten different connections can be drawn between any two dots of the five-dot pattern (S1 File). These connections can be combined into 1023 different designs; each correct design is a combination of one or more connections. Therefore, the first step of the software was to identify all correct, or true, connections in a cell. This was done by a set of algorithms that performed a series of subsequent tasks for each cell of the standard RFFT protocol:

1. identifying the active dots in each cell (Fig 2).
2. identifying the candidate connections (Fig 2).
3. designating all red pixels of the design drawn by the respondent to candidate connections.
4. checking if the red pixels that are designated to a specific candidate connection actually form a line that is compatible with the candidate connection. If not, the candidate connection is rejected; if so, the candidate connection undergoes the next check.
5. checking if the candidate connection is a false positive error. If so, the candidate connection is rejected; if not, the candidate connection is accepted as a true connection (Fig 2).

After performing task 1 to 5, the software combined the true connections that were identified in a cell into one design and calculated a design identifier (design ID). The design IDs, which were exclusive and corresponded to only one of the 1023 correct designs, were used to count the number of unique designs and perseverative errors. Further details on the software can be found in the supporting information (S1 File).

### Statistical analysis

Normally distributed data are presented as mean and standard deviation (SD) and non-normally distributed data as median and interquartile range (IQR). Differences in continuous data were tested with the unpaired t-test or, if appropriate, Mann-Whitney U-test. Differences in proportions were tested with the Chi-squared test. Agreement between two human assessments as well as between the computerized and human assessment was analyzed by the two-way mixed, absolute agreement, single measures intraclass correlation coefficient (ICC) and Lin’s concordance correlation coefficient. In addition, 95% limits of agreement were calculated by the Bland-Altman method. In all analyses, the level of statistical significance was set at 0.05. Lin’s concordance correlation coefficients were calculated with Stata Statistical Software Release 13 (StataCorp LP, College Station, TX, USA). All other statistical analyses were done with IBM SPSS Statistics 22.0 (IBM, Armonk, NY, USA).

## Results

### Study population

The total study population included 1,761 persons (Table 1): 948 men (54%) and 813 women (46%). The mean age (SD) was 58 (10) years. Ten percent of the study population had completed 0 to 8 years of education: International Standard Classification of Education (ISCED) level 0–1 [13]; 26%, 9 to 12 years: ISCED level 2; 28%, 13 to 15 years: ISCED level 3–4; and 36%, 16 years or more: ISCED level 5. The mean number of unique designs (SD) was 81 (29) and the median number of perseverative errors (IQR) was 9 (4–16), according to human assessment (criterion standard).

**Table 1:** Characteristics of the study population.

N (%)	1761 (100)
**Sex, n (%)**	
Men	948 (54)
Women	813 (46)
**Mean age (SD), years**	58 (10)
**Age groups, n (%), years**	
40–49	453 (26)
50–59	596 (34)
60–69	444 (25)
≥70	268 (15)
**Education, n (%), years^a^**	
0–8	169 (10)
9–12	452 (26)
13–15	494 (28)
≥16	638 (36)
Missing	8 (1)
**RFFT performance^b^**	
Unique designs, mean (SD)	81 (29)
Perseverative errors, median (IQR)	9 (4 to 16)

Abbreviations: IQR, interquartile range; RFFT, Ruff Figural Fluency Test; SD, standard deviation.

^a^ percentage does not add to 100 due to rounding.

^b^ according to human assessment (criterion standard).

### Agreement between human assessments

#### Unique designs

**Fig 3:**
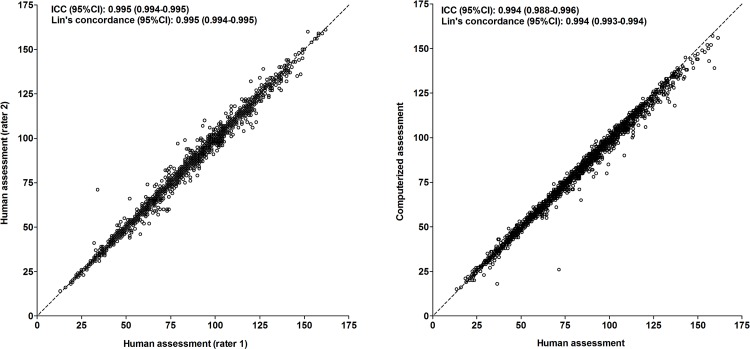
Comparison of computerized and human assessment of the number of unique designs. Left: human vs. human assessment (independent raters); right: computerized vs. human assessment. Broken lines represent lines of identity. Abbreviations: ICC, intraclass correlation coefficient; Lin’s concordance, Lin’s concordance correlation coefficient.

**Fig 4:**
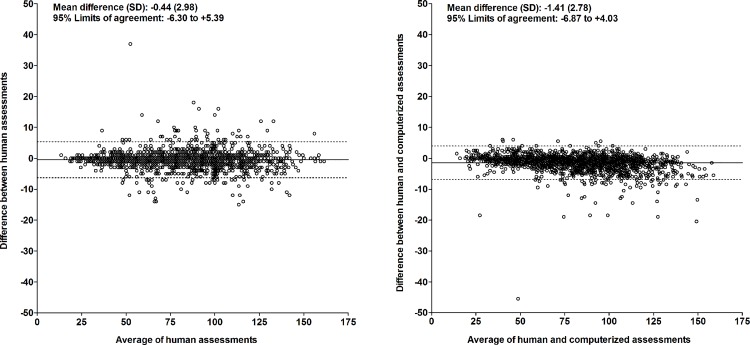
Bland-Altman plot of computerized and human assessment of the number of unique designs. Left: human vs. human assessment (independent raters); right: computerized vs. human assessment.

For the number of unique designs, the intraclass correlation coefficient between two human assessments (different raters) was 0.995 (95%CI, 0.994 to 0.995; p<0.001)(Fig 3, left panel); the Lin’s concordance correlation coefficient was 0.995 (95%CI, 0.994 to 0.995; p<0.001). The mean difference (SD) between two human assessments (different raters) was -0.44 (2.98). This was not dependent on the average result of the assessments (Fig 4, left panel). The 95% limits of agreement were -6.30 and +5.39.

#### Perseverative errors

**Fig 5:**
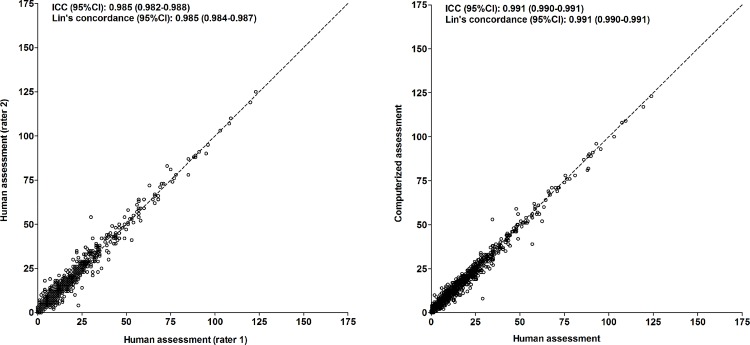
Comparison of computerized and human assessment of the number of perseverative errors. Left: human vs. human assessment (independent raters); right: computerized vs. human assessment. Broken lines represent lines of identity. Abbreviations: ICC, intraclass correlation coefficient; Lin’s concordance, Lin’s concordance correlation coefficient.

**Fig 6:**
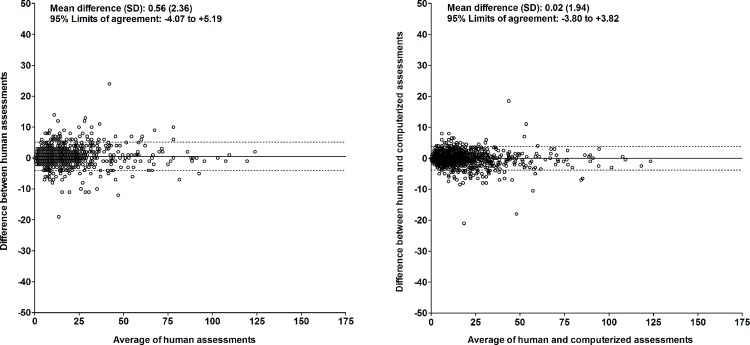
Bland-Altman plot of computerized and human assessment of the number of perseverative errors. Left: human vs. human assessment (independent raters); right: computerized vs. human assessment.

For the number of perseverative errors, the intraclass correlation coefficient between two human assessments (different raters) was 0.985 (95%CI, 0.982 to 0.988; p<0.001)(Fig 5, left panel); the Lin’s concordance correlation coefficient was 0.985 (95%CI, 0.984 to 0.987; p<0.001). The mean difference (SD) between two human assessments (different raters) was +0.56 (2.36). This was not dependent on the average result of the assessments (Fig 6, left panel). The 95% limits of agreement were -4.07 and +5.19.

### Agreement between computerized and human assessment

#### Unique designs

For the number of unique designs, the intraclass correlation coefficient between the computerized and human assessment was 0.994 (95%CI, 0.988 to 0.996; p<0.001)(Fig 3, right panel); the Lin’s concordance correlation coefficient was 0.994 (95%CI, 0.993 to 0.994; p<0.001). The mean difference (SD) between the computerized and human assessment was -1.41 (2.78). This was dependent on the average result of the computerized and human assessment (Fig 4, right panel). The number of unique designs was somewhat higher in the computerized assessment than in the human assessment in persons with a low performance and somewhat lower in persons with a high performance. The 95% limits of agreement were -6.87 and +4.03.

There was one clear outlier in the comparison between the computerized and human assessment (Fig 3, right panel; Fig 4, right panel). According to the computerized assessment, the number of unique designs was 26 for this person but according to the human assessment, it was 72 (difference, -46 points). Visual inspection of the original RFFT protocol revealed that this person did not strictly follow the instructions when performing the RFFT. The lines that were drawn to connect the dots of the five-dot pattern of the RFFT did not merely connect the dots but went a few millimeters through and crossed the dots (S1 Fig). According to the computerized assessment, there were 49 violations of the procedure by this person. Most of these violations were assessed as a unique design by the human raters.

#### Perseverative errors

For the number of perseverative errors, the intraclass correlation coefficient between the computerized and human assessment was 0.991 (95%CI, 0.990 to 0.991; p<0.001)(Fig 5, right panel); the Lin’s concordance correlation coefficient was also 0.991 (95%CI, 0.990 to 0.991; p<0.001). The mean difference (SD) between the computerized and human assessment was +0.02 (1.94). This was not dependent on the average result of the computerized and human assessment (Fig 6, right panel). The 95% limits of agreement were -3.80 and +3.82.

## Discussion

The RFFT is a nonverbal fluency test that is sensitive to changes in cognitive function in young as well as old persons [4–6]. Therefore, the RFFT is a useful tool for life course studies of cognitive disorders as it is generally assumed that changes in cognitive function begin at a relatively young age [3]. However, conventional assessment of performance on the RFFT by human raters can be time-consuming and may be impractical in large sample studies. Therefore, we developed a dedicated software program for computerized assessment of the performance on the RFFT to be able to assess the performance of a large number of persons in a relatively short time. In this study, we report that the agreement between the computerized and human assessment was very high and comparable to the agreement between two independent human assessments. The computerized and human assessments yielded highly concordant results. This makes the software program well-suited for the assessment of performance on the RFFT in other large sample studies.

The agreement between two independent human assessments of performance on the RFFT, or interrater reliability, was investigated in only a few studies that included relatively small or highly specific populations. In a study by Berning *et al*., 143 RFFT protocols were assessed by 30 pairs of raters [14]. They found an ICC of 0.93 for unique designs and 0.74 for perseverative errors. In a study by Sands, 50 RFFT protocols of patients with mixed neurological disease were assessed by two raters [15]. Sands found an ICC of 0.99 for unique designs and 0.99 for perseverative errors. Finally, in a study by Ross *et al*., 90 RFFT protocols of healthy young persons, undergraduate students recruited from introductory psychology courses, were all assessed by seven raters [16]. They found an ICC of 0.95 for unique designs and 0.86 for perseverative errors. Thus, in all three studies, it was found that the agreement between independent human raters was high to very high for the number of unique designs and moderate to high for the number of perseverative errors. For the number of unique designs, these results were confirmed in our study as we also found a very high agreement between human raters for this measure. For the number of perseverative errors, however, we also found a very high agreement. This difference between the other studies and our study can probably be explained by the much larger study population in our study. Furthermore, there was a difference between the other studies and our study in source population. Whereas the other studies included persons from highly specific source populations such as patients with neurological disease or students, our study included community-dwelling persons ranging in age from 40 years to 75 years or older, and ranging in educational level from primary school to university. Therefore, it can be concluded that the RFFT has a high to very high interrater reliability and that this finding is not limited to specific study samples but can be generalized to the adult population.

The assessment of RFFT protocols can be difficult and time-consuming because persons who undergo the test may draw complicated designs or a large number of highly similar designs. This is particularly the case in study samples that include young and highly educated people [6]. As a consequence, assessment of performance on the RFFT can be challenging and requires fine visual discrimination and sustained attention to detail [14]. Not surprisingly, the accuracy of the assessment is dependent on the rater’s experience of assessment as well as on his own performance on the RFFT [14]. Although the effects of these two factors are probably small, their impact can be substantial in large scale studies or over a large number of clinical cases [14]. To avoid these sources of error and to be able to assess a large number of RFFT protocols as part of the Lifelines Cohort Study [12], we developed dedicated software for the computerized assessment of the RFFT. In this study, the software performed well and we found a high agreement between the computerized and human assessment. For the number of perseverative errors, the agreement between the computerized and human assessment was even somewhat higher than the agreement between two independent human raters. Thus, the software that we developed for computerized assessment of performance on the RFFT is an accurate tool that can reduce time and manpower needed for the assessment of RFFT protocols in large scale studies.

The mean difference between the computerized and human assessment of performance on the RFFT was quite small and comparable to the mean difference between two independent human assessments. Nevertheless, in 5% of the participants, the difference was more than five unique designs or more than three perseverative errors and in a smaller percentage of participants, these differences were even much higher. Although this probably is of minor importance in large scale studies, we think that these differences can be relevant for the assessment of individual patients in clinical practice. However, similar differences were found between independent human assessments. In our study, such differences were partly explained by differences in the interpretation of the scoring rules as specified in the professional manual [5], and not so much by overseeing erroneous designs or perseverative errors. Due to the time pressure that is part of the RFFT, participants may work hastily and draw lines that are not straight but curved or lines that do not completely connect the dots of the five-dot pattern. Such imprecise designs may be assessed differently by different raters. Here, computerized assessment probably is more consequent and reproducible than human assessment although clearly, the accuracy of computerized assessment is also dependent on adherence to the test instructions.

The importance of adherence to the test instructions was further underlined by the finding that the number of unique designs was slightly underestimated in the computerized assessment as compared with the human assessment. It has been our experience that participants who work fast and draw curved lines or lines that do not completely connect the dots are mostly people with high performance on the RFFT. Probably, human raters are more liberal and consider such hasty designs more often as correct designs than the computer software. Although it can be debated whether human assessment was too liberal or computer software too strict, it is likely that the agreement between human and computerized assessment can be still further improved by strict adherence to the test instructions and, if necessary, repeated feedback during administration of the RFFT. We also recommend that a statement along the lines of "when drawing a design, make sure each line extends all the way to the dot" is included in the standard instructions for the RFFT.

A potential limitation to our study is the recruitment and training of the human raters. Although we recruited young and highly educated people as raters and all raters received training and supervision, the raters in our study were not professional neuropsychologists or psychometrists. Possibly, the agreement between professional neuropsychologists and psychometrists is higher than the agreement between the human assessments reported in this study. It may also be higher than the agreement between the computerized and human assessments. On the other hand, young and highly educated people generally have the best performance on the RFFT [6], and the raters in this study gained extensive experience in the assessment of RFFT protocols. Both rater fluency and performance have a positive effect on the assessment accuracy of RFFT protocols [12]. The main strength of our study is its study population that included a large number of community-dwelling persons who varied widely in age and educational level. As a result, the agreement between the computerized and human assessments could be studied across a wide performance range which is important for the generalizability of our findings.

## Conclusion

In this study, the agreement between two independent human assessments, or interrater reliability, of performance on the RFFT was very high. We also found that the agreement between the computerized and human assessment was very high and comparable to the agreement between the human assessments. Thus, in large scale studies, performance on the RFFT can be accurately assessed by the software application specifically designed for this task.

## Supporting Information

S1 Fig RFFT part 1 form of the outlier in the comparison between the computerized and human assessment of the number of unique designs. Difference between computerized and human assessment, -46 points.
(TIF)

S1 File Computerized assessment of performance on the Ruff Figural Fluency Test (RFFT): basic principles and algorithms. (DOCX)

## References

[1] Ferri CP, Prince M, Brayne C, Brodaty H, Fratiglioni L, Ganguli M, et al Global prevalence of dementia: a Delphi consensus study. Lancet. 2005:366: 2112–2117. 10.1016/S0140-6736(05)67889-0 16360788 PMC2850264

[2] Prince M, Bryce R, Albanese E, Wimo A, Ribeiro W, Ferri CP. The global prevalence of dementia: a systematic review and metaanalysis. Alzheimers Dement. 2013;9: 63–75.e2. 10.1016/j.jalz.2012.11.007 23305823

[3] Singh-Manoux A, Kivimaki M, Glymour MM, Elbaz A, Berr C, Ebmeier KP, et al Timing of onset of cognitive decline: results from Whitehall II prospective cohort study. BMJ. 2012;344: d7622 10.1136/bmj.d7622 22223828 PMC3281313

[4] Ruff RM, Light RH, Evans RW. The Ruff Figural Fluency Test: a normative study with adults. Dev Neuropsychol. 1987;3: 37–51. 10.1080/87565648709540362

[5] Ruff RM. Ruff Figural Fluency Test: professional manual Lutz: Psychological Assessment Resources, Inc.; 1996.

[6] Izaks GJ, Joosten H, Koerts J, Gansevoort RT, Slaets JP. Reference data for the Ruff Figural Fluency Test stratified by age and educational level. PLoS One. 2011;6: e17045 10.1371/journal.pone.0017045 21347325 PMC3037396

[7] Fama R, Sullivan EV, Shear PK, Cahn-Weiner DA, Marsh L, Lim KO, et al Structural brain correlates of verbal and nonverbal fluency measures in Alzheimer's disease. Neuropsychology. 2000;14: 29–40. 10.1037/0894-4105.14.1.29 10674796

[8] Foster PS, Williamson JB, Harrison DW. The Ruff Figural Fluency Test: heightened right frontal lobe delta activity as a function of performance. Arch Clin Neuropsychol. 2005;20: 427–434. 10.1016/j.acn.2004.09.010 15896557

[9] Stolk RP, Rosmalen JG, Postma DS, de Boer RA, Navis G, Slaets JP, et al Universal risk factors for multifactorial diseases: Lifelines: a three-generation population-based study. Eur J Epidemiol. 2008;23: 67–74. 10.1007/s10654-007-9204-4 18075776

[10] Lambers Heerspink HJ, Brantsma AH, de Zeeuw D, Bakker SJ, de Jong PE, Gansevoort RT, et al Albuminuria assessed from first-morning-void urine samples versus 24-hour urine collections as a predictor of cardiovascular morbidity and mortality. Am J Epidemiol. 2008;168: 897–905. 10.1093/aje/kwn209 18775924

[11] Mahmoodi BK, Gansevoort RT, Veeger NJ, Matthews AG, Navis G, Hillege HL, et al Microalbuminuria and risk of venous thromboembolism. JAMA. 2009;301: 1790–1797. 10.1001/jama.2009.565 19417196

[12] Strauss E, Sherman EMS, Spreen O. Ruff Figural Fluency Test. In: A compendium of neuropsychological tests: administration, norms, and commentary 3rd ed. New York: Oxford University Press; 2006 pp. 466–471.

[13] UNESCO Institute for Statistics. International Standard Classification of Education: ISCED 2011. Montreal: UNESCO Institute for Statistics; 2012.

[14] Berning LC, Weed NC, Aloia MS. Interrater reliability of the Ruff Figural Fluency Test. Assessment. 1998;5: 181–186. 10.1177/107319119800500208 9626393

[15] Sands KA. (1997) Nonverbal fluency: a neuropsychometric investigation. (Order No. 9803816, Saint Louis University). ProQuest Dissertations and Theses. Retrieved from http://search.proquest.com/docview/304366414?accountid=11219. (304366414).

[16] Ross TP, Foard LE, Hiott FB, Vincent A. The reliability of production strategy scores for the Ruff Figural Fluency Test. Arch Clin Neuropsychol. 2003;18: 879–891. 10.1016/S0887-6177(02)00163-4 14609582

