# Supplementary figures and images for: Agreement between Computerized and Human Assessment of Performance on the Ruff Figural Fluency Test
Source: PLoS One. 2016 Sep 23;11(9):e0163286. doi: 10.1371/journal.pone.0163286 (PMC5035016; doi:10.1371/journal.pone.0163286)

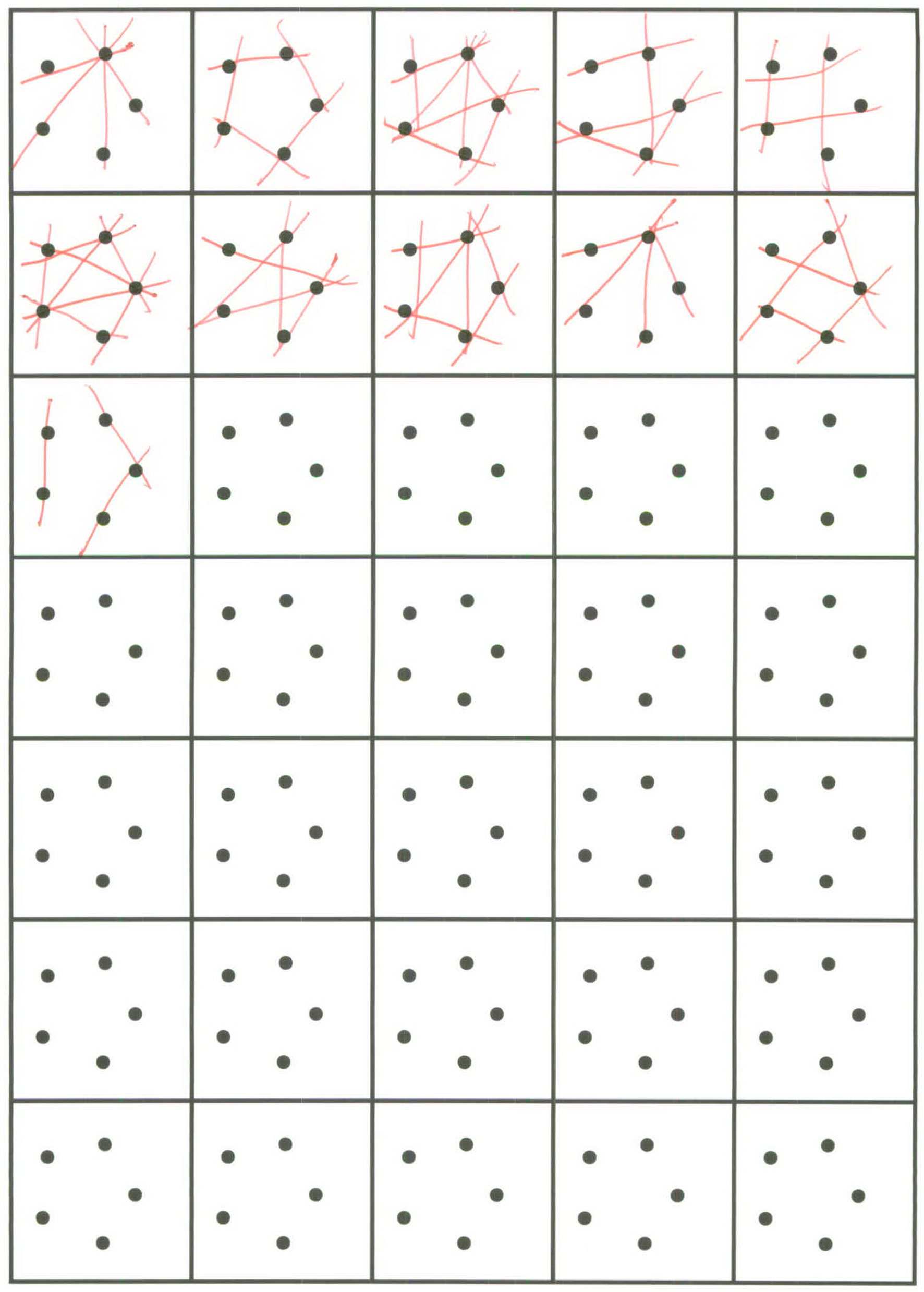

Supplement: S1 Fig — Difference between computerized and human assessment, -46 points. (TIF) [file pone.0163286.s001.tif]
