# Supplementary material for: Agreement between Computerized and Human Assessment of Performance on the Ruff Figural Fluency Test
Source: PLoS One. 2016 Sep 23;11(9):e0163286. doi: 10.1371/journal.pone.0163286 (PMC5035016; doi:10.1371/journal.pone.0163286)
Supplement: S1 File — (DOCX) [file pone.0163286.s002.docx]

**S1 File. Computerized assessment of performance on the Ruff Figural Fluency Test (RFFT): basic principles and algorithms.**

**Administration of the RFFT**

The Ruff Figural Fluency Test (RFFT) was administered to respondents according to the instructions that are detailed in the professional manual [2]. For the computerized assessment, it is essential that the respondent uses a red pencil with a pencil width of minimal 1 mm or, ideally, between 3-5 mm.

**Basic principle**

Development of the software was based on the principle that in each cell of the standard RFFT protocol, no more than ten unique connections can be drawn between two dots (Fig 1). These connections can be combined into 1023 different (unique) designs; each (correct) design is a combination of one or more connections. (Strictly speaking, there are 1024 different unique designs but the design with no connections is considered as an empty cell.)

**
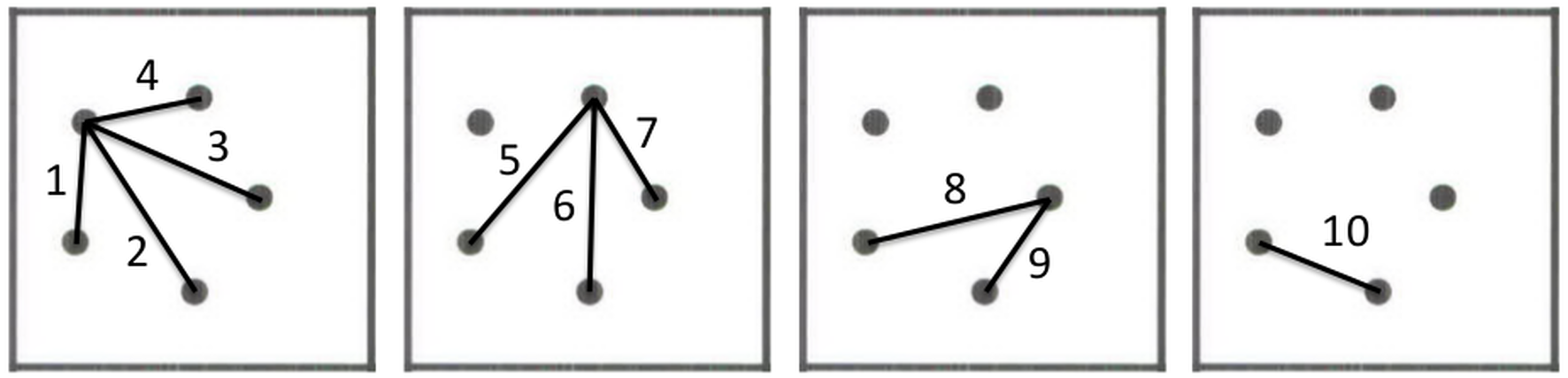
Fig 1. The ten unique connections that can be drawn between two dots in one cell of the standard RFFT protocol.** Each design is a combination of one or more of these connections.

**Algorithms to recognize designs and assess performance**

The recognition of designs is performed by a set of algorithms. These algorithms execute a series of subsequent tasks for each cell of the standard RFFT protocol.

1. identifying the active dots in each cell (Fig 2).

2. identifying candidate connections (Fig 2).

3. designating all red pixels of the design drawn by the respondent to candidate connections.

4. checking if the red pixels that are designated to a specific candidate connection actually form a line that is compatible with the candidate connection. If not, the candidate connection is rejected; if so, the candidate connection undergoes the next check.

5. checking if the candidate connection is a false positive error. If so, the candidate connection is rejected; if not, the candidate connection is accepted as a true connection (Fig 2).

**
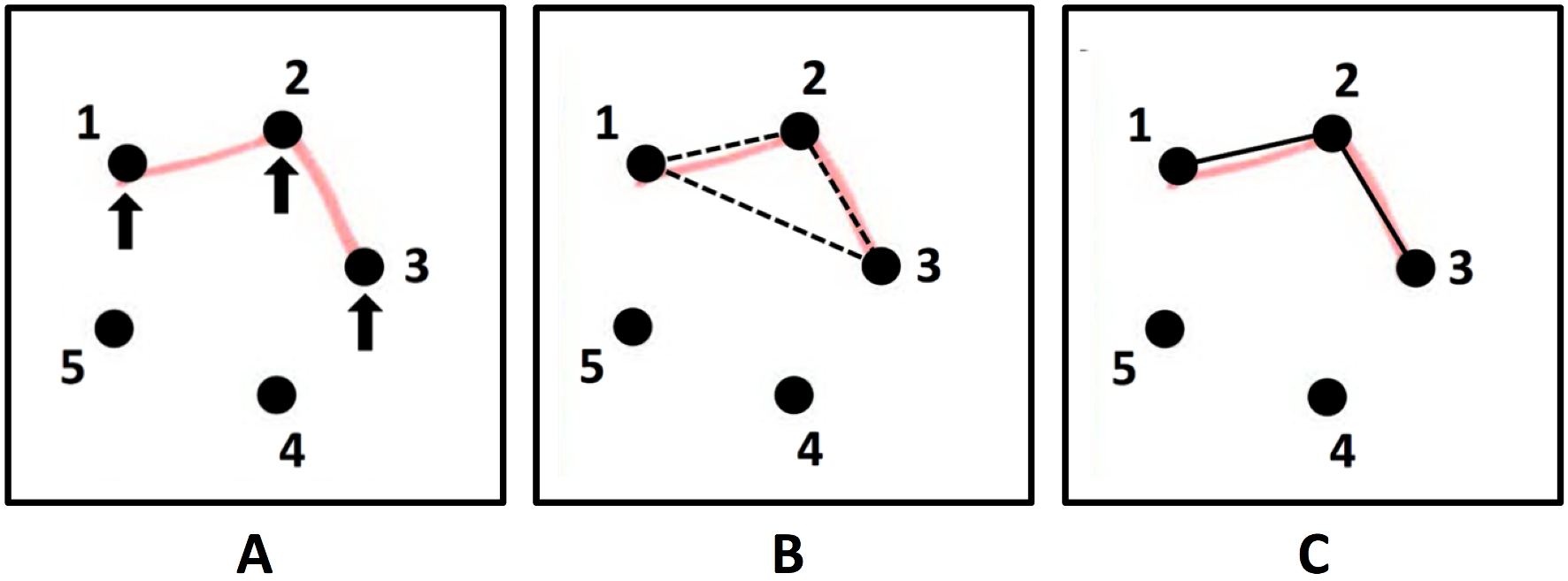
Fig 2. Illustration of terminology.** A. Active dots are part of the design drawn by the respondent (red lines). Here, 1, 2, and 3 are active dots (arrows); 4 and 5 are inactive dots. B. Candidate connections are all connections that can possibly exist between the active dots (dashed black lines). C. True connections are connections that are compatible with the red lines drawn by the respondent: here, connections 1–2 and 2­­­­–3 (black lines).

After performing task 1 to 5 for each cell of the standard RFFT protocol, the software performs one additional task.

6. counting the number of designs by checking the true connections in each cell and calculating a design identifier (design ID). Design IDs are exclusive and correspond to only one of the 1023 unique designs that can be drawn. The designs and design IDs are included in a design library that is part of the software.

These tasks are described in more detail in the following sections.

**Identification of active dots**

First, an algorithm distinguishes between active and inactive dots in each cell of the standard RFFT protocol. Active dots are part of the design and connected by the red lines drawn by the respondent in a specific cell (Fig 2). Inactive dots are not a part of the design. Dots are identified as active dots if there are any red pixels close to it (within a certain radius). Dots are identified as inactive if there are no red pixels around it because this makes it highly unlikely that the dot is part of a design drawn by the respondent and connected to one of the other dots.

Identification of active dots can be difficult in part 3 of the standard RFFT protocol because in this part, each cell contains black lines to distract the respondent. Therefore, an extra image processing routine was added to the software program. This routine removes all black lines from the cells while preserving the dots of the five-dot pattern.

**Identification of candidate connections**

Once the active dots have been identified, it is clear which are the candidate connections (Fig 2). By definition, the candidate connections are all connections that can be drawn between the active dots in a cell; connections between an active and inactive dot, or between two inactive dots cannot be candidate connections as it is highly unlikely that inactive dots are part of a design drawn by the respondent. In the next steps, the software evaluates which of the candidate connections are true connections and which must be rejected.

**Designating red pixels**

The first step in further evaluating whether a candidate connection is a true connection or must be rejected is designating the red pixels that are part of the design drawn by the respondent to one or more of the candidate connections. This is done by a voting algorithm that compares the coordinates of every red pixel detected in a specific cell to the coordinates of the candidate connections. If the coordinates of the red pixel are within the (predefined) boundaries of a candidate connection, the red pixel is designated as a part of this candidate connection. Dependent on the position, direction and curviness of the red lines drawn by the respondent, red pixels can be designated as a part of more than one candidate connection.

**Checking compatibility designated pixels and candidate connection**

The next step is to evaluate whether the red pixels that are designated as part of a candidate connection are actually compatible with this connection. This is done by algorithms that check whether the line formed by the red pixels is continuous and long enough. If this line is broken or too short, the red pixels were erroneously designated as part of this candidate connection and belong to another candidate connection. Erroneously designating red pixels as part of a specific candidate connection can be the result of hastily and inaccurate drawing by the respondent.

Additional algorithms calculate several other characteristics of the line formed by the red pixels such as the distance between end points, and the average and maximum distance between the line and the ideal position of the candidate connection (Fig 1, 2). These values are further used to evaluate whether the candidate connection is a true connection or must be rejected.

**Checking for false positive errors**

Nevertheless, some false positive errors may occur and the algorithm may identify true connections between two dots that have not been connected by the respondent (Fig 3).


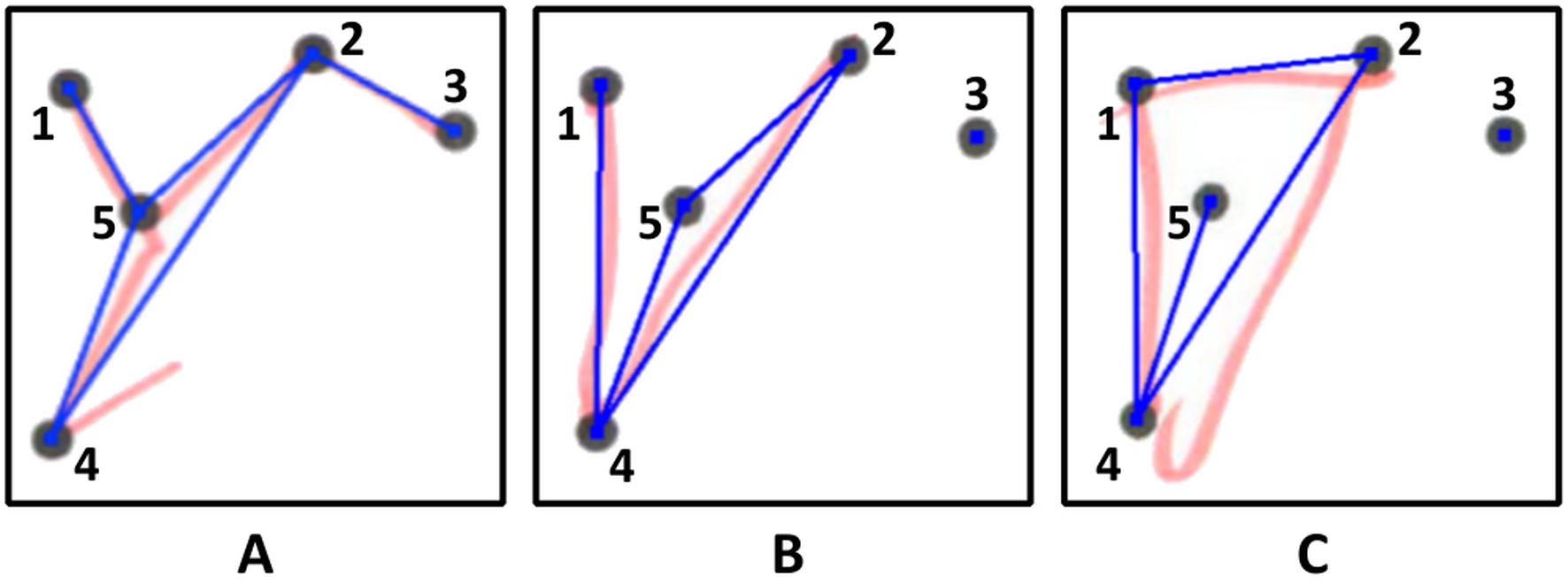
**Fig 3. Examples of erroneously identified true connections (false positive errors).** A. the connection 2–4. B. the connections 2–5 and 4–5. C. the connection 4–5. In all examples, the red line that was drawn by the respondent is close to the ideal position and direction of more than one (candidate) connection. As a result, the algorithm erroneously identifies more true connections.

Most of these false positive errors can be prevented by an extra analysis of the identified candidate connections. This analysis includes a triangles filter and pairs filter. Both filters use the average distance between the red line drawn by the respondent and the candidate connections as well as the coordinates of the end points of the red line. The triangles filter performs the analysis for candidate connections that form a triangle (for example, the candidate connections 2–5, 2–4, and 4–5 in Fig 3A), and the pairs filter for candidate connections that share a dot and form a pair (for example, the candidate connections 1–4 and 4–5 in Fig 3C).

**Counting the number of designs**

The final step is to count the number of designs that are drawn by the respondent. For each part of the standard RFFT, the algorithm checks the true connections in all 35 cells and calculates a design ID. Design IDs are exclusive and correspond to only one unique design. For empty cells, the design ID is 0; for cells with violations, the design ID is -1. The algorithm counts the number of unique design (n_u_), empty cells (n_e_), and violations (n_v_) and calculates the number of perseverative errors (35–n_u_–n_e_–n_v_).

**Developers and contributors**

The software was developed by Sander Pham and Martin F. Elderson with contributions by Johan Kok, Melanie M. van der Klauw, and Bruce H.R. Wolffenbuttel, Groningen University, University Medical Center Groningen, Lifelines Cohort Study and Department of Endocrinology, Groningen, the Netherlands.

This text was written by Gerbrand J. Izaks with contributions by Sander Pham and Martin F. Elderson.

**Contact**

m.f.elderson@umcg.nl (MFE), b.h.r.wolffenbuttel@umcg.nl (BHRW)

**References**

1. Stolk RP, Rosmalen JG, Postma DS, de Boer RA, Navis G, Slaets JP, et al. Universal risk factors for multifactorial diseases: LifeLines: a three-generation population-based study. Eur J Epidemiol. 2008;23: 67-74. doi: 10.1007/s10654-007-9204-4
2. Ruff RM. Ruff Figural Fluency Test: professional manual. Lutz: Psychological Assessment Resources, Inc.; 1996.
